# Looking a bit superficial to the pleura

**DOI:** 10.1186/s13089-014-0013-1

**Published:** 2014-08-22

**Authors:** Pablo Blanco, Giovanni Volpicelli

**Affiliations:** 1 Intensive Care Unit, Hospital Dr. Emilio Ferreyra, 4801, 59 St, Necochea 7630, Argentina; 2 Emergency Medicine, San Luigi Gonzaga University Hospital, Turin 10043, Italy

**Keywords:** Ultrasonography, Internal thoracic vessels, Pericardiocentesis, CABG

## Abstract

**Background:**

The internal thoracic artery (ITA) is a descendant branch of the subclavian artery. The former is located bilaterally in both internal sides of the thorax near the sternum and is accompanied by two internal thoracic veins (ITV). From a practical point of view, the ITA (and the ITV) identification is important because these vessels can be injured when pericardiocentesis with the parasternal approach is used. Other advantage of the ITA recognition is to check the patency of the ITA grafts in coronary artery revascularizated patients with new onset chest pain. The purpose of this article is to introduce a simple ultrasonographic technique for recognition of the aforementioned vessels and to highlight the utility of this finding in clinical practice.

**Findings:**

With linear probe and along paraesternal line, the internal thoracic vessels are recognized on grayscale imaging as an anechoic tubular structure immediately anterior to pleural line. Color Doppler identifies a pulsatile (ITA) and a non-pulsatile (ITV) flow. Spectral Doppler normally shows a high resistance velocity profile in non-grafted ITA and a phasic flow in ITV. A biphasic low resistance velocity profile is normally expected in the grafted and permeable ITA.

**Conclusions:**

The ITA (non-grafted) and ITV are recognized routinely along the parasternal line. The operators should identify these vessels when the parasternal approach pericardiocentesis is required and should also consider obtaining spectral Doppler images to check permeability of grafted ITA in coronary artery bypass graft patients with chest pain.

## Findings

**Figure 1:**
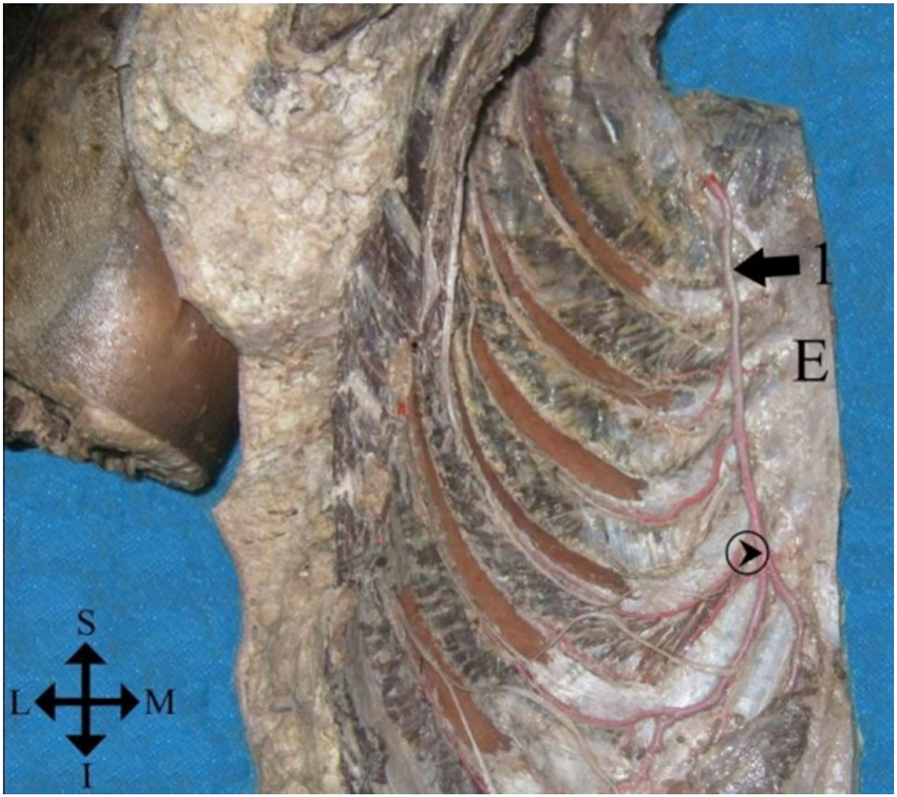
**Cadaveric endothoracic view.** Hemi-left anterior chest wall. E, sternum; 1, internal thoracic artery; circle with arrow inside, ITA bifurcation. S, superior; L, lateral; M, medial; I, inferior [1].

The internal thoracic artery (ITA; also known as internal mammary artery) is a prescalenic collateral descendant branch of the subclavian artery. Its origin is medial with respect to the phrenic nerve (PN), and it is posterior regarding the venous brachiocephalic trunk (VBT). The ITA descends and reaches the lower edge of the VBT, where the PN is anteromedially located, and then the ITA is directed forward in search of the medial border of the first rib, where it is folded in an oblique inward journey to reach the vicinity of the lateral edge of the sternum, where it becomes downward again. In most cases, at the level of the sixth rib cartilage, it is divided into its two terminal branches: the superior epigastric and musculophrenic arteries (Figure 1) [1]. The ITA is accompanied by two internal thoracic veins (ITV; also known as internal mammary veins) which ascend medially and laterally, respectively, and drains in the subclavian vein [2].

The importance of the ITA (specially the left artery) is largely recognized because it is often used for coronary artery bypass grafting (CABG) to bypass the left anterior descending or the circumflex artery [3].

Another aspect of the ITA (and the ITV) is the possibility of needlestick injury when pericardiocentesis is practiced with the parasternal approach. This one can be especially necessary in anterior located pericardial effusions [4], when the largest pericardial fluid accumulation is closest to the chest wall [5],[6] and finally when a longer path exists to reach the pericardial fluid from the subxifoid approach [5]. Possible complications related to the ITA injury are pseudoaneurysm formation [7], ITA fistulas [6],[8], bleeding into pericardium and mediastinum, and significant blood loss [4].

Although the occurrence of these complications have not been studied comparatively with the blind technique versus the ultrasound-guided procedure, in the large study of Tsang et al. [9], the ITA puncture was reported in none of 122 ultrasound-guided pericardiocentesis performed by the parasternal approach (89 left parasternal, 39 right parasternal). Nagdev et al. [10] reported that the ITA is rarely in the path of the needle when performing the in-plane parasternal long technique for pericardiocentesis.

In a study of 60 embalmed specimens of anterior thoracic walls obtained from adult Indian cadavers of both sexes, it was observed that the ITA (both the right and left) near their terminal end approaches closer towards the lateral sternal border and suggests precautions while performing emergency pericardiocentesis, preferably using real-time ultrasound guidance [11].

Finally, the ITA identification in post-CABG patients with new onset chest pain adds the possibility to check the permeability of the graft and assess coronary flow reserve [2],[3],[12]–[14].

### Ultrasound technique for location of ITA and ITV

Previous publications described how to locate the ITA with phased array ultrasound transducers, especially when this vessel is used in CABG, usually bypassing the anterior descending artery [2],[3],[12]–[14]. Actually, with the use of thoracic ultrasound and high-frequency transducers, the ITA and the ITV are seen routinely along the parasternal line.

In two publications [4],[10], the location of the ITA was described with high-frequency linear transducers but the technique, ultrasound anatomy, and Doppler findings were not extensively explained.

**Figure 2:**
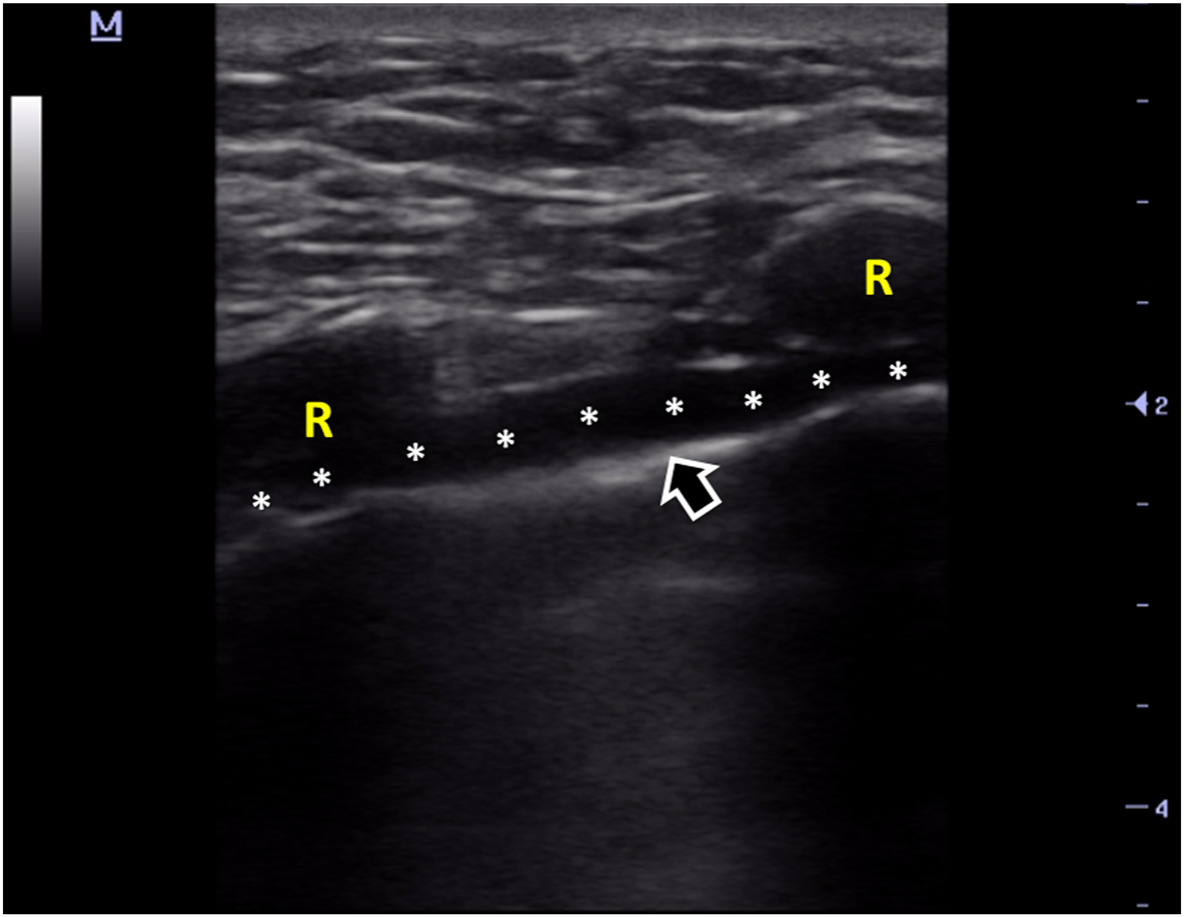
**Internal thoracic vessels identification.** Asterisks, internal thoracic vessels. R, ribs. Arrow, pleural line.

**Figure 3:**
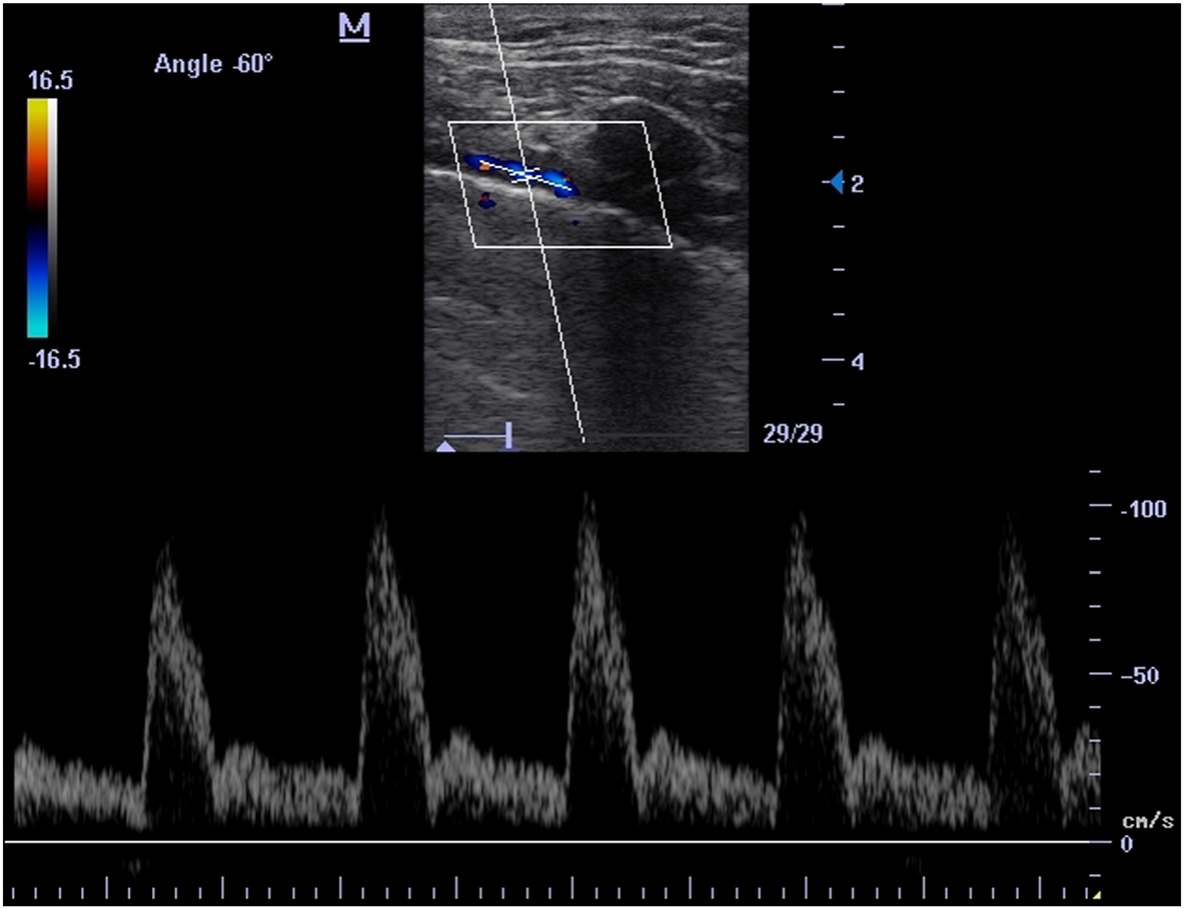
**PW of non-grafted ITA.** Note the high resistance velocity profile.

**Figure 4:**
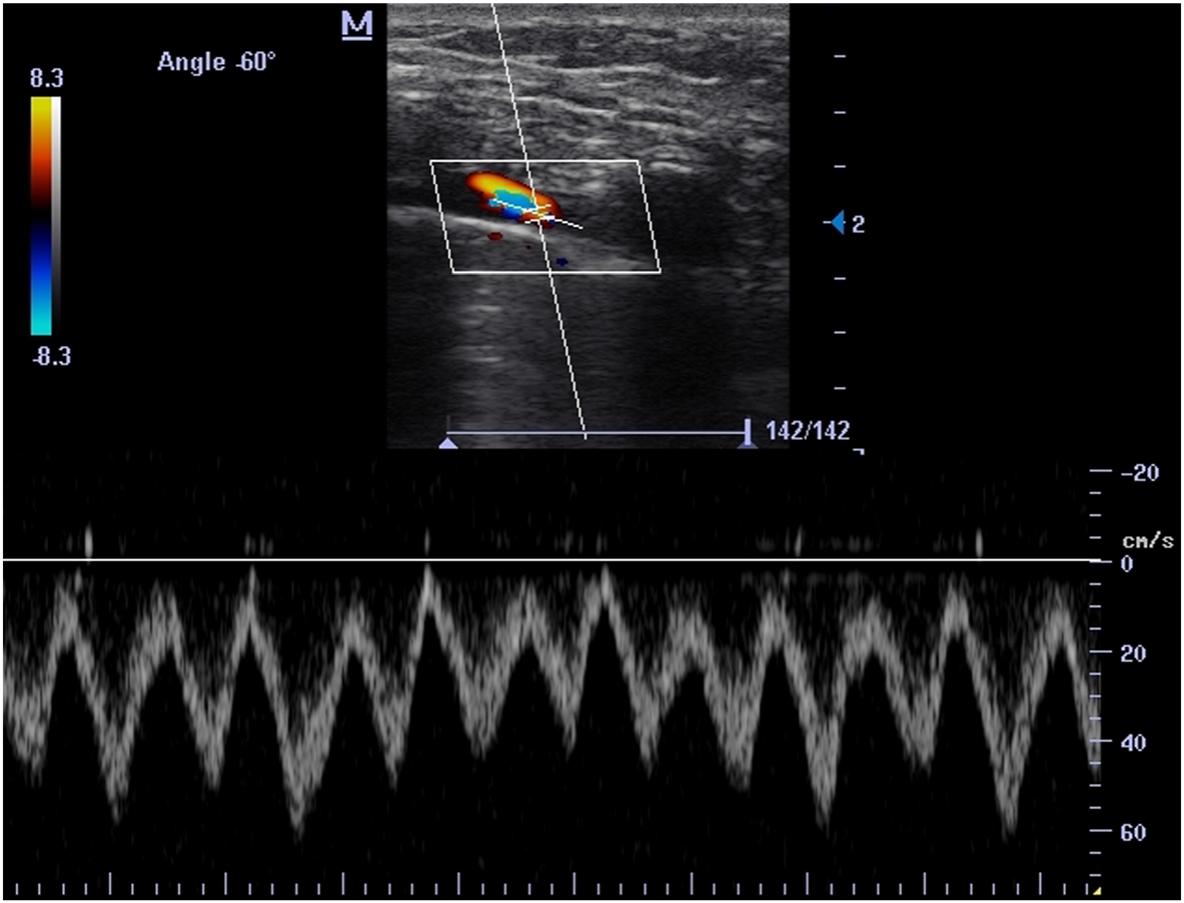
**PW of ITV.** Phasic flow with transmitted pulsatility of the near ITA.

Technically, with linear probe (5 to 10 MHz) and in longitudinal view, following the pleural line (PL) from anterolateral to near the sternum (Additional file 1: Clip 1) in most of the cases, an anechoic tubular structure is superficially appreciated, immediately anterior to PL (Figure 2, Additional file 2: Clip 2). This corresponds to the internal thoracic vessels. From cephalic to caudal, the extent of these vessels is evident between the ribs and anterior to PL (Additional file 2: Clip 2). At color Doppler, a pulsatile (artery) or non-pulsatile (vein) flow can be seen (Additional file 3: Clip 3 and Additional file 4: Clip 4). When pulsed wave spectral Doppler (PW) is used (Figure 3), a high resistance velocity profile can be detected in the non-grafted artery (unless subclavian artery does not have a hemodynamically significant stenosis) and a phasic flow is shown in permeable ITV with transmitted pulsatility of the near ITA (Figure 4). In grafted and permeable ITA, a low resistance biphasic with predominantly diastolic flow is normally seen [2]–[4],[12]–[14].

Due to the current technology of ultrasound transducers, it is possible to correctly find these vessels with convex probes, at the highest frequency allowed by the transducer, normally at 5 MHz. It is noteworthy that resolution is not always optimal compared to linear probe.

### Commentaries

There are no studies published to date addressing the feasibility of ultrasound for the recognition of the ITA and ITV along the parasternal line; nonetheless, in our clinical experience, non-grafted internal thoracic vessels are recognized in the total number of patients with the previously described technique. In grafted ITA, the accuracy of the described ultrasound technique is not the same for the reason that the native course of the ITA is roughly modified by the surgical procedure [2]. This unknown accuracy of ultrasound along the parasternal line for the ITA recognition can be a good point for future research in centers with high volume of CABG patients with the grafted ITA.

According to the author's knowledge, there are no studies focused to the learning curve of the internal thoracic vessel recognition with the ultrasound technique. Due to the fact that the aforementioned technique is a simple modification of the widely described thoracic ultrasound method for the recognition of pneumothorax, it could be speculated that this learning curve is the same ‘steep learning curve’ described in the literature for pneumothorax diagnosis [15].

### Conclusion

The internal thoracic vessels are recognized routinely in all patients examined with high-frequency ultrasound transducer along the parasternal line. Puncture of these vessels cannot be a real complication of pericardiocentesis (parasternal approach) if they are properly recognized with ultrasound. Another potentiality of ultrasound identification of the ITA is to, non-invasively, assess the permeability of the ITA after CABG in addition to the evaluation of coronary flow reserve.

## Abbreviations

ITA: internal thoracic artery

PN: phrenic nerve

VBT: venous brachiocephalic trunk

ITV: internal thoracic veins

CABG: coronary artery bypass grafting

PL: pleural line

PW: pulsed wave spectral Doppler

## Competing interests

The authors declare that they have no competing interests.

## Authors’ contributions

PB observed the cases and collected the videos and conceived and wrote the manuscript. GV contributed to the writing the manuscript. Both authors read and approved the final manuscript.

## Additional files

## Supplementary Material

Additional file 1: Clip 1. Technique for localization of the internal thoracic vessels. Click here for file

Additional file 2: Clip 2. Identification of the internal thoracic vessels superficial to the pleural line. Click here for file

Additional file 3: Clip 3. Color Doppler identification of the ITA. Click here for file

Additional file 4: Clip 4. Color Doppler identification of the ITV. Click here for file
